# Polysaccharides and polyphenol in dried *Morinda citrifolia* fruit tea after different processing conditions: Optimization analysis using response surface methodology

**DOI:** 10.7717/peerj.11507

**Published:** 2021-05-26

**Authors:** Qingfen Wang, Fei Yang, Dandan Jia, Tian Wu

**Affiliations:** Key Laboratory for Forest Resources Conservation and Utilization in the Southwest Mountains of China, Ministry of Education, Southwest Landscape Architecture Engineering Research Center of National Forestry and Grassland Administration, Kunming, Yunnan, China

**Keywords:** *Morinda citrifolia*, Polysaccharides, Polyphenols, Aqueous extraction, Response surface methodology

## Abstract

The increasing popularity of *Morinda citrifolia* has many medical and health benefits because of its rich polysaccharides (PSC) and polyphenols (PPN). It has become popular to brew the dry *M. citrifolia* fruit slice as tea in some regions of China. In this study, optimize the extraction parameters of *M. citrifolia fruit* tea polysaccharides and polyphenols using response surface methodology. The results indicated the highest PSC yield of 17% at 46 °C for 11 min and the ratio of water/*M. citrifolia* fruit powder was 78 mL/g. The optimum extraction of PPN was at 95 °C for 10 min and the ratio of water/*M. citrifolia* fruit powder 90 mL/g, with 8.93% yield. Using dry *M. citrifolia* fruit slices as a tea is reported for the first time. Based on the results, the maximum level of PSC can be obtained under condition by infusing about four dried *M. citrifolia* fruit slice with average thickness and size in warm boiled water for 11 min, taking a 300 mL cup (300 mL of water) for example. The maximum level of PPN can be obtained by adding three slices of dried *M. citrifolia* fruit slice to boiled water for 10 min. Considering the powder used in our study, the further pulverization of cutting into powder is more conducive to material precipitation. This study provides a scientific basis for obtaining PSC and PPN from dry *M. citrifolia* fruit slice tea by brewing.

## Introduction

*Morinda citrifolia* L. (Rubiaceae) was commonly referred to as noni in Hawaii, mengkudu in Malaysia, cheese fruit in Australia, Indian mulberry in India and also noni in China, and famous for its extensive therapeutic effects (Chan-Blanco et al., 2006; Jia, Lan & Wu, 2020; Motshakeri & Ghazali, 2015; Rajaei-Sharifabadi et al., 2017). As a traditional folk medical plant, especially the fruit is the most attractive part containing the most valuable chemical compounds (Assi et al., 2017; Lin et al., 2013; Mukti et al., 2019; Prasad, Visagaperumal Zonoubia & Chandy, 2019). Many studies have shown that noni has many functions such as anti-oxidation, anti-virus, anti-inflammatory, anti-cancer, anti-tumor, anti-injury, anti-bacterial, and scavenging free radicals. These effects continued believed to be related to polysaccharides and polyphenols (Nayak & Mengi, 2010; Santos & De Aquino Santana, 2019; Sasmito et al., 2015; Sousa et al., 2018).

Plant polysaccharides are one of the functional components in the *M. citrifolia* fruit (Sousa et al., 2018). It not only participates in energy metabolism, but also has a variety of biological activities, such as antiviral, antioxidant, anti-tumor, anticoagulant, etc., showing good commercial development prospects functional foods and medicine (Chen et al., 2011). Polysaccharide from lotus plumule can be used to treat type I diabetes and inflammation (Liao, Guo & Lin, 2011; Liao & Lin, 2011). The polysaccharides contained in lotus leaf and Pouteria campechiana seeds have a strong ability to scavenge free radicals, which may be a potential natural antioxidant resource (Zhang et al., 2015; Ma et al., 2019). Plant polyphenols are a kind of secondary metabolites, which had antibacterial, anti-inflammatory and antioxidant activities (Cai et al., 2004; Scalbert, Johnson & Saltmarsh, 2005; Darvesh et al., 2010), and were the main antioxidant in *M. citrifolia* (Srinivasahan & Durairaj, 2014). Grape is one of the fruits with the highest content of polyphenols. It is beneficial to health to absorb phenolic compounds from grape((Iglesias-Carres et al., 2018)). Extracts of *D. Morbifera* leaf are rich in polyphenols, which have antioxidant, anti-inflammatory, anti-obesity, anticancer activities and immunomodulatory functions ((Hyun et al., 2013; Hyun et al., 2015; Birhanu et al., 2018; Kang et al., 2018)). Studies have found that polyphenols are abundant in plants such as tea, thyme, rosemary and other plants (Favre et al., 2018; Favreau-Farhadi, Pecukonis & Barrett, 2015; Lunceford & Gugliucci, 2005). Plant extracts rich in polyphenols have the advantage of being a natural ingredient and are more acceptable than synthetic compounds (Peng et al., 2008). The naturally fermented *M. citrifolia* juice contains polyphenolic and polysaccharide, which is the main bioactive ingredient in *M. citrifolia* fruit (Motshakeri & Ghazali, 2015; Chang et al., 2013). The polysaccharides in *M. citrifolia* fruit can regulate the immune response, thereby significantly inhibiting tumor growth in human cancer patients (Sharma et al., 2015; Dussossoy et al., 2011). The potential use of *M. citrifolia* fruit as a health promoter caught the attention of food industry and the pharmaceutical sector made it as a part of various products. Currently, several methods have been used to reduce the unpleasant taste and smell of ripe *M. citrifolia* fruit, such as *M. citrifolia* juice, fermented juice, *M. citrifolia* syrup, *M. citrifolia* puree, *M. citrifolia* powder and *M. citrifolia* extract for medicines and chemical reagents (Almeida, De Oliveira & Hotza, 2019; Assi et al., 2017).

Tea is a time-honored and very acclaimed beverage that originated in China. It is made by young leaves and/or young stems of *Camellia sinensis* plant in a narrow sense (Kröppl et al., 2012; Taylor, Hamilton-Miller & Stapleton, 2005), and by leaves, flowers and fruit from any plant in a broad sense (Pekal, Biesaga & Pyrzynska, 2013). *Chrysanthemum morifolium* flowers (Guzelmeric, Vovk & Yesilada, 2015), flowers or flower buds of *Lonicerae Japonicae* Flos (Cai et al., 2020), the flower buds of Rosa (Chang et al., 2019) and the leaf of *Lycium barbarum* L. (Zhao et al., 2020) have been widely used as tea. Now there is a various unique tea in different places such as Teng Cha (China), Apple tea (Turkey), Chamomile tea (Russia and Germany), Mate tea (Argentina), Rooibos tea (South Africa) (Jin et al., 2016; Raal et al., 2012). Tea is a source of beneficial compounds that can supply nutrients for people (Cavuldak et al., 2019). Hot water could dissolve or extract vitamins, polyphenols, polysaccharides, and other plant bioactive compounds. For example, drinking magnolia tea could improve sleep quality and adjunct to postpartum depression (Xue et al., 2020). Mate tea containing numerous active compounds was benefited to the cardiovascular system (Heck & De Mejia, 2007). Moreover, chamomile tea had many potential health benefits because of its phenolic compounds, which have antioxidant and antibacterial effects (Mckay & Blumberg, 2006). Green tea could also prevent cancer, cardiovascular disease and other diseases consequent on its high content of phenolic compounds (Lorenzo & Munekata, 2016; (Sinija & Mishra, 2008)). The *M. citrifolia* fruit is universally used by fermenting, but it is more expensive and tastes average. Under the global enthusiasm for tea drinking, making *M. citrifolia* fruit tea with dried slices or powder is a fabulous option, and also a cheap, effective and convenient way, providing its active ingredients as polysaccharides and polyphenols for people. Because *M. citrifolia* fruit has a special smell when it is ripe, people generally do not eat fresh *M. citrifolia* fruit directly, but would further process it into *M. citrifolia* enzyme, *M. citrifolia* juice, *M. citrifolia* fruit wine, etc. The processing process of these products is complicated and the cost is high. In this study, *M. citrifolia* fruit slices were used to make tea, which not only saves cost but also is convenient to eat. This was the first time that the dried *M. citrifolia* fruit was used as a tea product, but how to infuse it to get more polysaccharides and polyphenols is still unclear. For instance, the water temperature, soaking time, and the ratio of water to dried *M. citrifolia* slices are unknown. Fruit tea’s resulting positive health effects would differ based on water temperature and infuse of the water-tea ratio (Şahin, 2013).

Response surface methodology (RSM) is an effective statistical method, which can predict the influence of the interaction between multiple independent variables on the dependent variables and find the optimal extraction conditions for experiments, thus significantly reducing the number of experiments, reducing the cost of experiments, and saving time (Zou et al., 2013; Silva, Rogez & Larondelle, 2007; Prasad et al., 2011). Box-Behnken design (BBD) and central composite design (CCD) are the most commonly used effective RSM techniques to optimize extraction conditions in complex extraction (Bezerra et al., 2008; Gardin et al., 2001; Han et al., 2001; Ding et al., 2016; Zhang et al., 2017; Cho & Zoh, 2007). Compared with CCD, the BBD program does not include extreme factor value combination (the highest or lowest level), which can avoid experiments under extreme factor value conditions and avoid possibly produce unsatisfactory results Li et al., 2019). In addition, the BBD method is the most economical and efficient design, this design requires a small number of runs, thus avoiding time-consuming experiments (Bezerra et al., 2008; Ferreira et al., 2007). Successfully optimized the extraction conditions of polysaccharides and polyphenols in a variety of plants using BBD-RSM, for instance, *Camellia sinensis* (Zheng et al., 2019), *Pistacia lentiscus* L. Leaves (Detti et al., 2020), olive leaves (Wang, Yang & Yang, 2020), *Actinidia chinensis* Planch. (Han et al., 2019), *Ziziphus jujuba cv*. (He et al., 2019). Therefore, based on single-factor experiments, RSM was used to carry out measurements and compare the contents of polysaccharides (PSC) and polyphenol (PPN) in dried *M. citrifolia* fruit slice tea with a dried slice under different process conditions including temperature, time and ratio of water/*M. citrifolia* fruit powder.

## Materials and Methods

### Overview of experimental program

**Figure 1:**
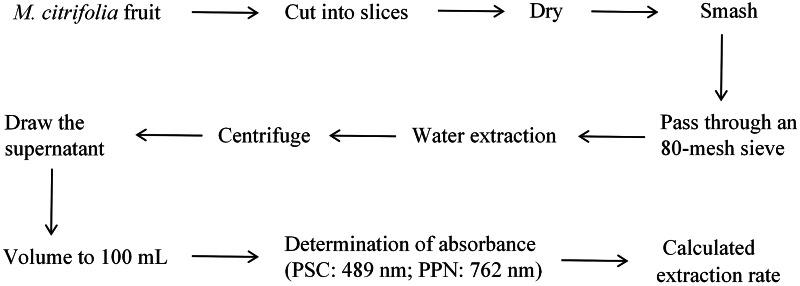
Schematic overview of the experimental program.

Wash the collected *M. citrifolia* fruit with distilled water, slice and dry them, grind them into powder, extract with water, and determine the content of PSC and PPN with an ultraviolet spectrophotometer. Figure 1 describes the experimented program.

### Collection of fruit samples

The *M. citrifolia* fruit was collected from plantations located Yuanjiang, Yunnan Province, China (25° 3″41.92″N, 102° 45″20.39″E). *M. citrifolia* was introduced by the research team from Hawaii in the United States in 2007 and planted in Yuanjiang plantations. *M. citrifolia* is a tropical plant, and its optimal growth temperature range is 20 to 30 °C (Gupta et al., 2020). Yuanjiang has a typical dry and hot valley climate, with an average annual temperature of 24.3 °C (Fei et al., 2017; Zhao et al., 2014). Collect *M. citrifolia* fruit in the white maturity stage and select fruit with the same maturity and size. Immediately after harvesting, immediately wash it carefully with distilled water to remove dust and other contaminants. Wipe off the residual moisture on the surface with absorbent paper for later use.

### Chemical and solvents

The chemicals used in this study, such as Folin-Ciocalteu reagent, gallic acid and glucose standard, were purchased in Sigma-Aldrich. All other solvents such as sulfuric acid, phenol and sodium carbonate were of analytical grade.

### Sample preparation and extraction

The *M. citrifolia* fruit was slice up of 0.5–0.8 mm and dried at 60 °C until the weight was constant, and grind it into a powder, pass through an 80-mesh sieve. The powder samples were put into a sealed bag and placed in a dryer at 60 °C for the later use. 0.5 g of *M. citrifolia* fruit powder was weighed, and explore the effect of different ratio of water/*M. citrifolia* fruit powder (70, 80 and 90 mL/g), extraction temperature (PSC: 40, 45 and 50 °C; PPN: 85, 90 and 95 °C) and extraction time (5, 10 and 15 min) on the extraction yield of PSC and PPN. The extraction solution was centrifuged at 3880 g for 10 min. The supernatant was used for the determination of PSC and PPN.

### Determination of PSC contents

The total PSC content of *M. citrifolia* extract was determined according to the phenol–sulfuric acid method (Qu et al., 2016) with modification using glucose as the standard. Each 0.1 mL sample added 0.9 mL distilled water and one mL of 5% phenol after mixed with 3.5 mL concentrated sulfuric acid rapidly. The maximal absorption was at 489 nm and the absorbance was measured after standing for 15 min.

The yield of PSC (%) was calculated in the same way as that of Qu et al. (2016).

### Determination of PPN contents

The total PPN content in the *M. citrifolia* extract was determined by using Folin–Ciocalteu method (Ab Rahman et al., 2019) with modification using gallic acid as the standard. Each 0.5 mL sample added 0.5 mL distilled water and one mL of Folin–Ciocalteu reagent, stood for 5 min. Then two mL of 10% sodium carbonate solution was added and stood about 1 h at room temperature in darkness. Finally, the maximal absorption at 762 nm was monitored.

The yield of PPN (%) was calculated in the same way as that of (Qu et al., 2016).

### Experimental design and data analysis

To determine the optimized aqueous extraction parameters of PSC and PPN, a Box-Behnken design (BBD) with three levels (−1, 0 and 1) was applied. Based on the single-factor results, the main scope of extraction parameters was identified. Table 1 showed the range and center points of the three operational parameters.

**Table 1:** The levels of parameters of BBD for extraction experimental.

Parameters		Codes	Levels		
			−1	0	1
PSC	Ratio of water/*M. citrifolia* fruit powder (mL/g)	X_1_	70	80	90
	Extraction temperature (°C)	X_2_	40	45	50
	Extraction time (min)	X_3_	5	10	15
PPN	Ratio of water/*M. citrifolia* fruit powder (mL/g)	X′_1_	70	80	90
	Extraction temperature (°C)	X′_2_	85	90	95
	Extraction time (min)	X′_3_	5	10	15

All data in this study were calculated as the average of experiments in triplicate. Response surface methodology (RSM) carried out the model and the experimental design and data analysis were implemented using Design-Expert software 8.0.6. To assess the statistical significance and the reliability of the model, response surface analysis and variance analysis (ANOVA) were performed. The statistically regarded was as significant if *P* < 0.05.

**Table 2:** Design and results of RSM experiment for extraction yield of PSC and PPN.

No.	Coded parameter levels of PSC			Coded parameter levels of PPN			Observer		Predicted	
	X_1_ (mL/g)	X_2_ (°C)	X_3_ (min)	X′_1_ (mL/g)	X′_2_ (°C)	X′_3_ (min)	Yield of PSC (%)	Yield of PPN (%)	Yield of PSC (%)	Yield of PPN (%)
1	0	1	−1	0	1	−1	14.46	7.8	14.40	7.80
2	−1	−1	0	−1	−1	0	15.05	7.4	14.89	7.49
3	1	−1	0	1	−1	0	15.19	8.4	15.19	8.39
4	0	0	0	0	0	0	16.92	8.6	16.93	8.53
5	−1	1	0	−1	1	0	16.10	8.0	16.10	8.01
6	0	0	0	0	0	0	16.84	8.6	16.93	8.53
7	1	0	1	1	0	1	15.56	8.9	15.51	8.91
8	−1	0	−1	−1	0	−1	15.50	7.8	15.56	7.79
9	1	1	0	1	1	0	15.19	8.3	15.35	8.21
10	0	1	1	0	1	1	15.81	8.5	15.71	8.58
11	1	0	−1	1	0	−1	15.59	8.3	15.49	8.39
12	0	0	0	0	0	0	17.02	8.4	16.93	8.53
13	−1	0	1	−1	0	1	15.80	8.5	15.90	8.41
14	0	−1	−1	0	−1	−1	14.74	7.9	14.84	7.83
15	0	−1	1	0	−1	1	13.84	8.2	13.90	8.20

## Results

### Model fitting analysis

The optimization of aqueous extraction parameter for high PSC extracts and PPN content from dried *M. citrifolia* fruit slice tea was conducted using RSM. The yield of PSC and PPN was considered a dependent variable for BBD. The experimental conditions of 15 runs were done respectively, with predicted results from PSC change interval of 13.84–17.02% and PPN change interval of 7.4−8.9% (Table 2). The regression equation of the predicted response Y for PSC and PPN yield was obtained as follows:

(1) \begin{eqnarray*}{Y}_{\mathrm{PSC}}(\text{%})& =-\mathrm{89}.\mathrm{52375}+\mathrm{0}.\mathrm{63058}{\mathrm{X}}_{1}+\mathrm{3}.\mathrm{49650}{\mathrm{X}}_{2}+\mathrm{0}.\mathrm{45367}{\mathrm{X}}_{3}-\mathrm{0}.\mathrm{00165}{\mathrm{X}}_{1}{\mathrm{X}}_{2}\nonumber\\\displaystyle & -\mathrm{0}.\mathrm{00525}{\mathrm{X}}_{1}{\mathrm{X}}_{3}+\mathrm{0}.\mathrm{0225}{\mathrm{X}}_{2}{\mathrm{X}}_{3}-\mathrm{0}.{\mathrm{0032208}}_{3}{\mathrm{X}}_{1}^{2}-\mathrm{0}.\mathrm{039683}{\mathrm{X}}_{2}^{2}-\mathrm{0}.\mathrm{048883}{\mathrm{X}}_{3}^{2}\end{eqnarray*}

(2) \begin{eqnarray*}{Y}_{\mathrm{PPN}}(\text{%})& =-\mathrm{24}.\mathrm{35}+\mathrm{0}.\mathrm{29417}{\mathrm{X}}_{1}^{{^{\prime}}}+\mathrm{0}.\mathrm{35750}{\mathrm{X}}_{2}^{{^{\prime}}}+\mathrm{0}.\mathrm{25083}{\mathrm{X}}_{3}^{{^{\prime}}}-\mathrm{0}.\mathrm{0005}{\mathrm{X}}_{1}^{{^{\prime}}}{\mathrm{X}}_{2}^{{^{\prime}}}\nonumber\\\displaystyle & -\mathrm{0}.\mathrm{0035}{\mathrm{X}}_{1}^{{^{\prime}}}{\mathrm{X}}_{3}^{{^{\prime}}}+\mathrm{0}.\mathrm{004}{\mathrm{X}}_{2}^{{^{\prime}}}{\mathrm{X}}_{3}^{{^{\prime}}}-\mathrm{0}.\mathrm{00116667}{\mathrm{X}}_{1}^{{^{\prime}}2}-\mathrm{0}.\mathrm{00166667}{\mathrm{X}}_{2}^{{^{\prime}}2}-\mathrm{0}.\mathrm{015667}{\mathrm{X}}_{3}^{{^{\prime}}2}\end{eqnarray*}

The ANOVA results determined the reliability of the model of PSC and PPN in predicting the optimum conditions and exactly displaying the real interrelationship between the selected parameters (Table 3).

**Table 3:** Variance analysis for fitting quadratic model of extraction yield of PSC and PPN.

Source	Sum of square	Degree of freedom	Mean square	*F*-value	*P*-value
Extraction of PSC					
Model	11.29	9	1.25	52.45	0.0002^**^
X_1_	0.11	1	0.11	4.42	0.0894
X_2_	0.065	1	0.065	2.71	0.1607
X_3_	0.94	1	0.94	39.24	0.0015^*^
X_1_X_2_	0.027	1	0.027	1.14	0.3348
X_1_X_3_	0.28	1	0.28	11.53	0.0194^*^
X_2_X_3_	1.27	1	1.27	52.93	0.0008^**^
X_1_^2^	0.38	1	0.38	16.02	0.0103^*^
X_2_^2^	3.63	1	3.63	151.97	<0.0001^**^
X_3_^2^	5.51	1	5.51	230.6	<0.0001^**^
Residual	0.12	5	0.024		
Lack of fit	0.1	3	0.034	4.23	0.197
Pure error	0.016	2	0.008133		
Correlation Total	11.41	14			
R^2^	0.9895	Adj R^2^	0.9707		
C.V.%	0.99	Pred R^2^	0.8519		
PRESS	1.69	Adeq Precision	23.991		
Extraction of PPN					
Model	2.09	9	0.23	16.76	0.0032^*^
X′_1_	0.6	1	0.6	43.73	0.0012^*^
X′_2_	0.66	1	0.66	47.8	0.001^**^
X′_3_	0.061	1	0.061	4.43	0.0893
X′_1_X′_2_	0.0025	1	0.0025	0.18	0.6884
X′_1_X′_3_	0.12	1	0.12	8.86	0.0309^*^
X′_2_X′_3_	0.04	1	0.04	2.89	0.1498
X′_1_^2^	0.05	1	0.05	3.63	0.115
X′_2_^2^	0.00641	1	0.00641	0.46	0.5263
X′_3_^2^	0.57	1	0.57	40.95	0.0014^*^
Residual	0.069	5	0.014		
Lack of fit	0.043	3	0.014	1.06	0.5183
Pure error	0.027	2	0.013		
Correlation Total	2.16	14			
R^2^	0.9679	Adj R^2^	0.9102		
C.V.%	1.43	Pred R^2^	0.6568		
PRESS	0.74	Adeq Precision	14.839		

**Notes.**

* Means significant at *P* < 0.05.

** Means significant at *P* < 0.001.

The regression coefficients were showed in Table 3. For PSC, X_3_, X_1_^2^, X_2_^2^, X_3_^2^, X_1_X_3_, X_2_X_3_were significant (*P* < 0.001). For PPN, X′_1_, X′_2_, X′_1_X′_3_, X′_3_^2^ was extremely significant (*P* < 0.001). In all the significant terms, the most significant effect on PSC and PPN were X_3_^2^ and X′_2_, respectively.

### Response surface analysis of PSC

**Figure 2:**
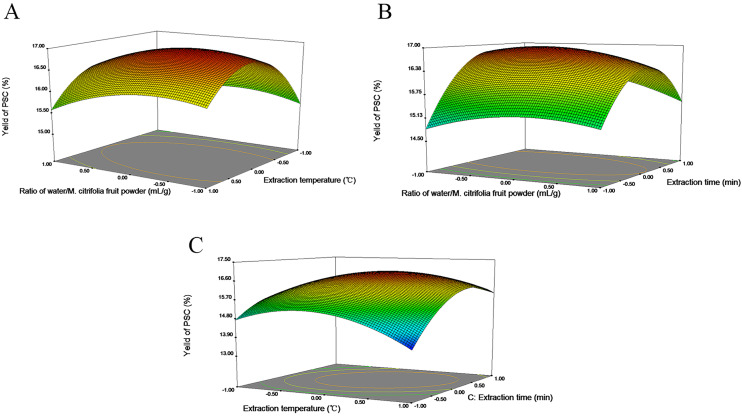
The 3D response surface plot and contour plot of PSC extraction yield. (A) Interaction between ratio of water/*M. citrifolia* fruit powder and temperature, while time was constant at 10 min; (B) interaction between ratio of water/*M. citrifolia* fruit powder and time, while the temperature was constant at 45 ° C; (C) interaction between temperature and time, while ratio of water/*M. citrifolia* fruit powder was constant at 80 mL/g).

The relationship between the response of PSC extraction condition was directly reflected by Fig. 2. At 40∼45 °C, PSC yield with the increase of extraction temperature, while the further addition of extraction temperature caused to decrease in the PSC yield (Fig. 2A). Moreover, under the extraction temperature of 45 °C and the ratio of water/*M. citrifolia* fruit powder of 80 mL/g, the extraction value could reach 17.02%. PSC yield was gradually increased by extension of extraction time but reduced at higher from 11 min extraction times. Moreover, up to 16.84% of PSC yield was obtained at an extraction time of 10 min and the ratio of water/*M. citrifolia* fruit powder of 80 mL/g (Fig. 2B).The extraction yield of PSC increases first and then decreases with the increase of extraction time and temperature (Fig. 2C). When the extraction time and temperature was 10 min and 45 °C, respectively, the maximum PSN yield was achieved.

### Response surface analysis of PPN

**Figure 3:**
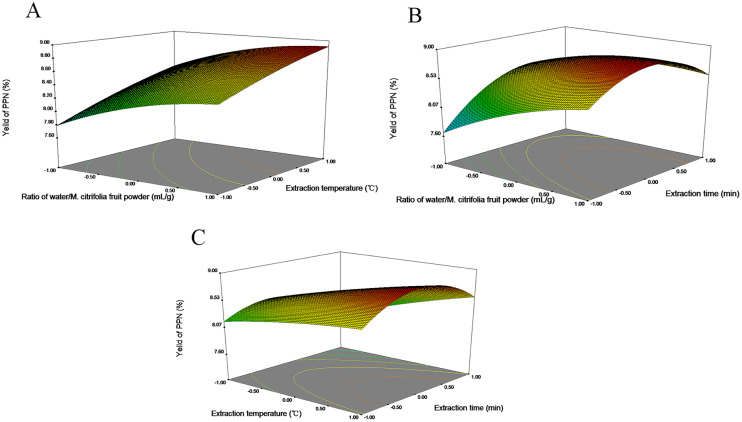
The 3D response surface plot and contour plot of PPN extraction yield. (A) Interaction between ratio of water/*M. citrifolia* fruit powder and temperature, while time was constant at 10 min; (B) interaction between ratio of water/*M. citrifolia* fruit powder and time, while the temperature was constant at 90 ° C; (C) interaction between temperature and time, while ratio of water/*M. citrifolia* fruit powder was constant at 80 mL/g).

The extraction yield of PPN increased with the extraction temperature and the ratio of water/*M. citrifolia* fruit powder (Fig. 3A). The optimal region was supposed to be in the ratio of water/*M. citrifolia* fruit powder above 80 mL/g, a temperature higher than 92 °C which could lead to the extraction yield of PPN over 8.8%. Furthermore, Fig. 3B illustrated that the ratio of water/*M. citrifolia* fruit powder was positively correlated with the extraction yield of PPN. An increase in the ratio of water/*M. citrifolia* fruit powder tend to enhanced the PPN yield. Moreover, the extraction time showed quadratic effects on the PPN extraction response. From 5 to 10 min, PPN yield showed an upward trend, and decline slightly over 10 min. Overall, the PPN showed the maximum yield when the ratio of water/*M. citrifolia* fruit powder was 90 mL/g for 10 min. The optimal region temperature and time of PPN extraction were about 91−95 °C and 8-15 min, respectively, and peaked at PPN yield when 95 °C and 11 min (Fig. 3C).

### Optimization conditions and model effectiveness

The optimal conditions for the PSC and PPN extraction were selected based on the constructed RSM models. The predicted yield of 16.98% could be obtained under the ratio of water/*M. citrifolia* fruit powder of 77.27 mL/g, the temperature of 45.56 °C and time of 10.98 min for PSC. The predicted yield of 8.91% could be obtained under the ratio of water/*M. citrifolia* fruit powder of 90 mL/g, the temperature of 95 °C and time of 10.09 min for PPN. To verify the effectiveness of models in predicting the yields of PSC and PPN, the conditions were modified based on the actual operation situation and experimental verification on optimized conditions. In the adjusted conditions, the practical yield of PSC and PPN was 17.00 ± 0.08 mg/g and 8.93 ±  0.04 mg/g, respectively (Table 4), which were approximately the predicted results, confirming that the response models were sufficient and precise for the prediction of PSC and PPN extraction.

**Table 4:** Results of experimental verification.

Response value	Optimal condition			Predicted value (%)	Experimental value (%)
	Ratio of water/ *M. citrifolia* fruit powder (mL/g)	Extraction temperature (° C)	Extraction time (min)		
Yield of PSC	78	46	11	16.98	17.00 ± 0.08
Yield of PPN	90	95	10	8.91	8.93 ± 0.04

## Discussion

*P*-values described the significance of the model and coefficients, and the adequacy and fitness of the model were checked into by the determination coefficient (R^2^) and the adjusted determination coefficient (Adj R^2^) (Zeng et al., 2020). The RSM mathematical model was significant (*P* < 0.05) for PSC and PPN did not reveal lack of fit (*P* > 0.05) (Table 3), which confirmed the model accuracy (Liu et al., 2019). According to with Adj R^2^ 0.9707 and 0.9102 for PSC and PPN, respectively, these models could sufficiently explain 97.07% and 91.02% response values and only about the change in 2.93%, 8.98% data could not be explained with these model. Moreover, exceptionally high values of R^2^ for PSC and PPN (0.9895 and 0.9679, respectively) and low values of the coefficient of variation (CV ) showed that the model was good (Hosseini et al., 2018). In the model, the term with a large F-value and a small *P*-value was taken as response variables, significantly impacting the extraction yield (Akhbari et al., 2018). To sum up, the experiment design is reasonable, which indicated that the RSM analysis method was feasible to optimize the extraction of PSC and PPN from dried *M. citrifolia* fruit slice tea.

The interactive effects of temperature on PSC yield were more substantial than the ratio of water/M. citrifolia fruit powder (Fig. 2A). This might result from the higher temperatures that could increase cell wall structure rupture, causing more PSC to dissolve in water. However, the macromolecules started to decomposition when the sensitive temperature to extract the PSC exceeded, resulting in less available PSC (Romdhane et al., 2017). On the other hand, the extraction time also showed a stronger effect on PSN yield in comparison to the ratio of water/noni fruit powder(Fig. 2B) . This might happen probably because of PSC properties during hydrolysis (Skenderidis et al., 2017; (Yang et al., 2013)). Therefore, the extraction temperature and time showed a more substantial effect on the PSC yield than the ratio of water/*M. citrifolia* fruit powder.

Figure 2C implied that the extraction time and temperature both had a strong effect on PSC yield and presented quadratic effects on the PSC extraction response, which verified that temperature and time (X_2_X_3_) in Table 3 were extremely significant. Generally, active substances’ solubility could be promoted by increasing extraction time and temperature to obtain a better extraction yield. Whereas, degradation of the active substances was also facilitated by prolonged extraction time and temperature, correspondingly leading to less yield (Garlock et al., 2011; Papoutsis et al., 2016). Ji et al. (2018) reported the similar findings that excessive temperature and time had a negative effect on the extraction yield, because the chemical structure and activity of PSC dissolved in water was influenced. Moreover, the semblable phenomenons were reported in studies by Lin et al. (2017), Shang et al. (2018) and Wang et al. (2014). The influence order of the interaction between response variables on the PSC yield is as follows: interaction of temperature and time (X_2_X_3_) > interaction of ratio of water/*M. citrifolia* fruit powder and time (X_1_X_3_) > interaction of ratio of water/*M. citrifolia* fruit powder and temperature (X_1_X_2_). This is consistent with the results of ANOVA (Table 3).

The extraction yield of PPN increased with the increase ratio of water/*M. citrifolia* fruit powder (Fig. 3A). The mass transfer principle might be considered, with the increase of ratio of water/*M. citrifolia* fruit powder, the concentration gradient in the solution raised, which meant that the driving force to dissolve the PPN in the water improved and the diffusion rate in the solution also enhanced (Al-Farsi & Lee, 2008; Goldsmith et al., 2014). The research showed that with the increase of liquid–solid ratio, the extraction rate of phenolic substances in citrus increased (Hayat et al., 2009). Our study showed that the high extraction temperature and high ratio of water/*M. citrifolia* fruit powder were favorable for the extraction of PPN when keeping time at the optimal values (Fig. 3A). The high temperature may promote the extraction yield by increasing the solubility and mass transfer rate of phenolic compounds (Dai & Mumper, 2010). Besides, the energetic conditions were higher when there was the higher temperature, leading to changes in solvent physical property and plant cell–matrix and an increase in the solubility and diffusion rate of PPN in water, which could be conducive to the extraction of PPN (Dos Santos Nascimento et al., 2018; Rajha et al., 2014; Zeković et al., 2014). The results of this experiments were similar to other studies on the optimization of water extraction of phenolics from lemon pomace, in which the extraction with the highest temperature was 95 °C and the highest sample-to-solvent ratio of 100 mL/g was the best yield of phenolics when compared with the yield obtained at the lower temperature and sample-to-solvent ratio (Papoutsis et al., 2016), and so did the study by Goldsmith et al. (2014). The influence order of the interaction between extraction conditions on the PPN yield is as follows: interaction of ratio of water/*M. citrifolia* fruit powder and time (X′_1_X′_3_) > interaction of temperature and time (X′_2_X′_3_) > interaction of ratio of water/*M. citrifolia* fruit powder and temperature (X′_1_X′_2_). This is consistent with the results of ANOVA (Table 3).

## Conclusions

To study how to drink dried *M. citrifolia* fruit slice tea to get more PSC and PPN. This research used RSM for obtaining the optimal conditions of the aqueous extraction process of PSC and PPN from *M. citrifolia* fruit, and the models were confirmed to be effective and feasible for optimization extraction. The optimal experimental conditions for extraction of PSC and PPN in dried *M. citrifolia* fruit slice tea were: the highest yield of PSC was 17.00% at ratio of water/*M. citrifolia* fruit powder 78 mL/g, 46 °C, and 11 min, and the highest yield of PPN was 8.93% at ratio of water/*M. citrifolia* fruit powder 90 mL/g, 95 °C, and 10 min. Thus, using a 300 ml cup (300 mL of water) as an example, people can soak four average-sized dried *M. citrifolia* fruit slices in boiled water for 11 min to obtained the maximum level of PSC. To get a PPN, people can place three slices in boiling water for 10 min. The design of this study was based on the respective optimizations of PSC and PPN. If people would like to obtain more PSC and PPN at the same time, the further experimental research is needed. The use of dried *M. citrifolia* fruit powder when conditions are permitted is more conducive to the precipitation of active substances than the dried slice. *M. citrifolia* fruit contains polysaccharides, polyphenols, alkaloids, amino acids and other compounds. It has antibacterial, anti-cancer, anti-oxidant, anti-inflammatory, analgesic, cardiovascular protection, etc. *M. citrifolia* fruit containing other active ingredients The optimal processing conditions for tea also needs to be further studied. The obtained findings promoted the development and utilization of *M. citrifolia* fruit products. It laid the foundation for further research on the functions of dried *M. citrifolia* fruit sliced tea.

##  Supplemental Information

10.7717/peerj.11507/supp-1 Supplemental Information 1 Raw data

## Additional Information and Declarations

### Data Availability

The following information was supplied regarding data availability: The raw date are available in the Supplementary File.
